# Abundant Microsatellite Diversity and Oil Content in Wild *Arachis* Species

**DOI:** 10.1371/journal.pone.0050002

**Published:** 2012-11-20

**Authors:** Li Huang, Huifang Jiang, Xiaoping Ren, Yuning Chen, Yingjie Xiao, Xinyan Zhao, Mei Tang, Jiaquan Huang, Hari D. Upadhyaya, Boshou Liao

**Affiliations:** 1 Key Laboratory of Biology and Genetic Improvement of Oil Crops, Ministry of Agriculture, Oil Crops Research Institute of the Chinese Academy of Agricultural Sciences, Wuhan, China; 2 National Key Laboratory of Crop Genetic Improvement and National Center of Plant Gene Research (Wuhan), Huazhong Agricultural University, Wuhan, China; 3 International Crops Research Institute for the Semi-Arid Tropics, Patancheru, Andhra Pradesh, India; Ben-Gurion University, Israel

## Abstract

The peanut (*Arachis hypogaea*) is an important oil crop. Breeding for high oil content is becoming increasingly important. Wild *Arachis* species have been reported to harbor genes for many valuable traits that may enable the improvement of cultivated *Arachis hypogaea*, such as resistance to pests and disease. However, only limited information is available on variation in oil content. In the present study, a collection of 72 wild *Arachis* accessions representing 19 species and 3 cultivated peanut accessions were genotyped using 136 genome-wide SSR markers and phenotyped for oil content over three growing seasons. The wild *Arachis* accessions showed abundant diversity across the 19 species. *A. duranensis* exhibited the highest diversity, with a Shannon-Weaver diversity index of 0.35. A total of 129 unique alleles were detected in the species studied. *A. rigonii* exhibited the largest number of unique alleles (75), indicating that this species is highly differentiated. AMOVA and genetic distance analyses confirmed the genetic differentiation between the wild *Arachis* species. The majority of SSR alleles were detected exclusively in the wild species and not in *A. hypogaea*, indicating that directional selection or the hitchhiking effect has played an important role in the domestication of the cultivated peanut. The 75 accessions were grouped into three clusters based on population structure and phylogenic analysis, consistent with their taxonomic sections, species and genome types. *A. villosa* and *A. batizocoi* were grouped with *A. hypogaea*, suggesting the close relationship between these two diploid wild species and the cultivated peanut. Considerable phenotypic variation in oil content was observed among different sections and species. Nine alleles were identified as associated with oil content based on association analysis, of these, three alleles were associated with higher oil content but were absent in the cultivated peanut. The results demonstrated that there is great potential to increase the oil content in *A. hypogaea* by using the wild *Arachis* germplasm.

## Introduction

The genus *Arachis* originated in South America and contains at least 80 species that have been classified into nine taxonomic sections based on morphological variation, geographical distribution and cross-compatibility [1], [2]. Some *Arachis* species are used as forage for animal production in Asia and Africa. The most economically important species in the genus is the cultivated peanut *Arachis hypogaea*. Peanuts are widely grown in more than 100 countries and are used to produce food and edible oil for human consumption. Because the amount of peanut oil used in developing countries is increasing, more than 60% of peanuts produced worldwide are crushed for edible oil (http://faostat.fao.org/faostat/collections?subset=agriculture 2010). Thus, enhancing the oil content of peanut cultivars is becoming an increasingly important breeding objective in most developing countries.

While most wild *Arachis* species are diploid (such as AA or BB, 2n = 2x = 20), the cultivated peanut is an allotetraploid (AABB, 2n = 4x = 40). A wild allotetraploid species in the *Arachis* section, *A. monticola* (AABB, 2n = 4x = 40), is thought to be the direct wild tetraploid ancestor of *A. hypogaea* [3], [4]. Cytological and molecular studies have shown that *A. duranensis* (AA) and *A. ipaënsis* (BB) have a high similarity to *A. hypogaea*. It is probable that *A. duranensis* and *A. ipaënsis* are the wild diploid progenitors of *A. hypogaea*, which may have arisen from a single hybridization event between *A. duranensis* and *A. ipaënsis* followed by chromosome duplication [3], [5]. Tetraploid species are also found in the sections *Extranervosae* and *Rhizomatosae*. These tetraploid species are believed to have evolved independently [6].

Wild *Arachis* species are widely distributed throughout a large region of South America and show extensive morphological variability depending upon their distinct environments. Perennial peanuts are characterized by tuberiform hypocotyls and tuberous roots for adaptation to upland areas (as in the sections *Erectoides* and *Extranervosae*). In contrast, annual species typically have fibrous root systems and reproductive systems adapted to lowland areas (as in the section *Arachis*) [7]. *Arachis* species are autogamous, disperse seeds underground and are geographically isolated from one another; because of these characteristics, hybrid sterility is often encountered when producing interspecific hybrids between species in the same section or in different sections [8]. This reproductive barrier among wild *Arachis* has inhibited the gene flow among these plants and may have facilitated the process of *Arachis* speciation. It is critical to pinpoint the genetic regions underlying the speciation process and determine the mechanism of divergent selection that shaped the different adaptive traits in wild *Arachis* species. Natural gene exchange between wild diploid species and cultivated peanut may have been further limited due to genomic re-arrangement during polyploidization [9]. In addition, domestication events have greatly reduced the genetic diversity in the cultivated peanut. Successive self-pollination and the use of a few elite breeding lines with little exotic germplasm in breeding programs resulted in a narrow genetic base of cultivated peanut germplasm [10], [11]. Thus, there is a potential to mine novel variants in wild species and transfer them into cultivated peanut. Wild *Arachis* species possess genetic variability in pest and disease resistance traits, which could be used to improve the cultivated peanut. These traits include resistance to peanut stunt virus (PSV) [12], peanut stripe virus (PStV) [13], nematodes [14], early leafspot [15], late leafspot [16], rust [4], bacterial wilt [17], and spotted spider mites [18]. The traits in some wild *Arachis* species that confer resistance to pests and disease have been successfully transferred into cultivated peanuts [19], [20].

High oil content in a high-yielding genetic background is a key objective of peanut breeding worldwide. Limited information is available regarding oil content variation in wild *Arachis* species and the relationship between oil content and genetic variation across the entire genome. Upadhyaya et al. reported that the range of oil content in wild *Arachis* accessions was 45%–55% at ICRISAT in Hyderabad, India [21]. Oil content is a quantitative trait controlled by many genes that have small effects and show high genotype × environment interactions. It is therefore of interest to investigate the variation in oil content among wild *Arachis* accessions in China and assess the value of these accessions in breeding. The characterization of the population structure and phylogenic relationships of wild *Arachis* accessions is essential to evaluate the level of differentiation among species and sections as well as to investigate the relationships between allelic variation and oil content in wild *Arachis* species. This information will be useful for identifying wild *Arachis* accessions that are ideal donor parents to enhance the cultivated peanut and to broaden the diversity of germplasm in peanut breeding.

In the present study, a collection of 3 cultivated peanut accessions and 72 wild *Arachis* accessions (representing 19 species from 5 different sections) was assembled. The *Arachis* collection was genotyped with 136 SSR primers and phenotyped for oil content. The objectives of the study were (a) to evaluate genetic diversity among accessions of different species and sections within the genus *Arachis*, (b) to infer the population structure and phylogenic relationship of the *Arachis* accessions and (c) to assess the variation of oil content in *Arachis* accessions and detect alleles associated with oil content.

## Results

### The Genetic Diversity of 75 *Arachis* Accessions

The 75 *Arachis* accessions used in this study belong to 20 species from 5 sections. These accessions were evaluated for allelic diversity using 136 SSR markers (Table 1). The wild *Arachis* accessions were highly diverse. In total, 944 alleles were identified (6.94 alleles per marker), and the Shannon-Weaver diversity index was 0.4130. Among the 19 wild *Arachis* species, *A. duranensis* exhibited the highest diversity, with a Shannon-Weaver diversity index of 0.3522. The cultivated *Arachis* (*A. hypogaea*) accessions exhibited relatively low diversity, with only 309 alleles identified (2.27 alleles per marker) and a Shannon-Weaver diversity index of 0.0662 (Table 1).

**Table 1:** Summary of genetic diversity and unique alleles among different *Arachis* species.

	Section	Species	N	Allele No.	Allele No./marker	Shannon-Weaver diversity index	Unique alleles
**Wild ** ***Arachis***	***Arachis***	***A. batizocoi***	4	558	4.10	0.29	3
		***A. cardenasii***	3	450	3.31	0.22	2
		***A. chacoense***	2	413	3.04	0.12	0
		***A. correntina***	3	451	3.32	0.17	1
		***A. duranensis***	23	750	5.51	0.35	12
		***A. helodes***	1	329	2.42	0.00	0
		***A. hoehnei***	2	421	3.10	0.17	2
		***A. kuhlmannii***	1	282	2.07	0.00	0
		***A. monticola***	6	532	3.91	0.20	3
		***A. stenosperma***	3	449	3.30	0.18	1
		***A. villosa***	6	517	3.80	0.19	5
	***Erectoides***	***A. cryptopotamica***	1	331	2.43	0.00	0
		***A. oteroi***	2	413	3.04	0.11	1
		***A. paraguariensis***	5	579	4.26	0.27	4
	***Heteranthae***	***A. pusilla***	2	406	2.99	0.14	4
	***Procumbentes***	***A. appressipila***	2	418	3.07	0.15	0
		***A. chiquitana***	1	310	2.28	0.00	0
		***A. rigonii***	4	535	3.93	0.28	75
	***Extranervosae***	***A. macedoi***	1	220	1.62	0.00	16
**Cultivated groundnut**	***Arachis***	***A. hypogaea***	3	310	2.28	0.07	3

### Unique Alleles Among *Arachis* Species and AMOVA

The number of unique alleles is an effective indicator of the genetic differentiation within a population [22]. Species-specific alleles were detected based on amplification events in different species to assess genetic differentiation. In total,129 unique alleles in different species were detected using 75 SSR markers (Table 1). This suggests that the wild *Arachis* accessions have high genetic diversity. Among these 75 SSR markers, 28 markers amplified multiple unique species-specific alleles, 25 markers were able to amplify alleles across all the 19 wild species. Different amplification events using the same SSR marker in different species reflected the distinct mutational histories of multiple alleles from the same microsatellite region. Different numbers of unique alleles were amplified in different species. *A. rigonii* exhibited the largest number of unique alleles (75), which suggested that *A. rigonii* has a high level of differentiation compared to other species. Several species, including *A. chacoense*, *A. helodes*, *A. kuhlmannii*, *A. cryptopotamica*, *A. appressipila* and *A. chiquitana*, did not exhibit any species-specific alleles, which may be because only a single accession was studied for each of these species.

AMOVA was performed to further investigate genetic differentiation among *Arachis* sections and species (Table 2). Approximately 7.34% (P<0.001) of the total molecular variation was attributed to genetic differentiation between the sections; 27.51% (P<0.001) was attributed to genetic differentiation among species within sections. This indicates that genus *Arachis* possesses wide diversity within both sections and species.

**Table 2:** Analysis of molecular variance among sections and species.

Source of variation	DF	Var component	Variation (%)
Among sections	4	10.92	7.34**
Among species withinsections	14	40.97	27.51**
Among accessions withinspecies	125	97.07	65.16**
Total	143	148.98	

** P<0.001, for 1000 permutations.

### The Alleles of Cultivated Peanut and its Wild Relatives

Determining the differences in genetic structure between *A. hypogaea* and its diploid and tetraploid wild relatives is necessary to understand the evolution of the cultivated peanut. Because the probable B-genome diploid wild progenitor *A. ipaënsis* was not included in this study, only *A. duranensis* (AA), *A. monticola* (AABB) and *A. hypogaea* (AABB) were considered in the analysis. In the comparison of *A. hypogaea* with its wild relatives, six evolutionary modes were described for microsatellite alleles during the domestication of cultivated peanut (Table 3): (i) Five alleles (0.66%) were amplified in *A. hypogaea* but not in *A. duranensis* and *A. monticola*, suggesting that these microsatellite regions emerged after the domestication of *A. hypogaea*; (ii) Eight alleles (1.05%) were amplified in *A. monticola* but not in *A. duranensis* and *A. hypogaea*. These alleles most likely emerged after the polyploidization event due to genomic rearrangements but were lost after the domestication event; (iii) Two hundred and ten alleles (27.63%) were amplified in *A. duranensis* but not in *A. monticola* and *A. hypogaea*. These alleles most likely emerged during the speciation of *A. duranensi*s but were lost after the polyploidization event; (iv) Two hundred and thirty-three alleles (30.66%) were amplified in *A. duranensis* and *A. monticola* but not in *A. hypogaea*. These alleles most likely emerged during the speciation of *A. duranensi*s but were lost in the domestication event; (v) Sixteen alleles (2.11%) were amplified in *A. duranensis* and *A. hypogaea* but not in *A. monticola*, indicating that the alleles most likely emerged independently in the speciation of *A. duranensis* and during the domestication of cultivated peanut; (vi) Two hundred and eighty-eight alleles (37.89%) were amplified in *A. duranensis*, *A. monticola* and *A. hypogaea*, indicating that these alleles were highly conserved during the evolution of cultivated peanut (Table 3).

**Table 3:** Summary of six evolutionary modes of microsatellite alleles amplified in the cultivated groundnut and its wild relatives.

Mode	*A. duranensis*	*A. monticola*	*A. hypogaea*	*n*	%
**i**	−	−	+	5	0.66
**ii**	−	+	−	8	1.05
**iii**	+	−	−	210	27.63
**iv**	+	+	−	233	30.66
**v**	+	−	+	16	2.11
**vi**	+	+	+	288	37.89

Note: “+” indicates that the SSR alleles are amplified in the specific species, “−” indicates that the SSR alleles aren’t amplified in the specific species.

### Population Structure and Phylogenic Analyses

**Figure 1:**
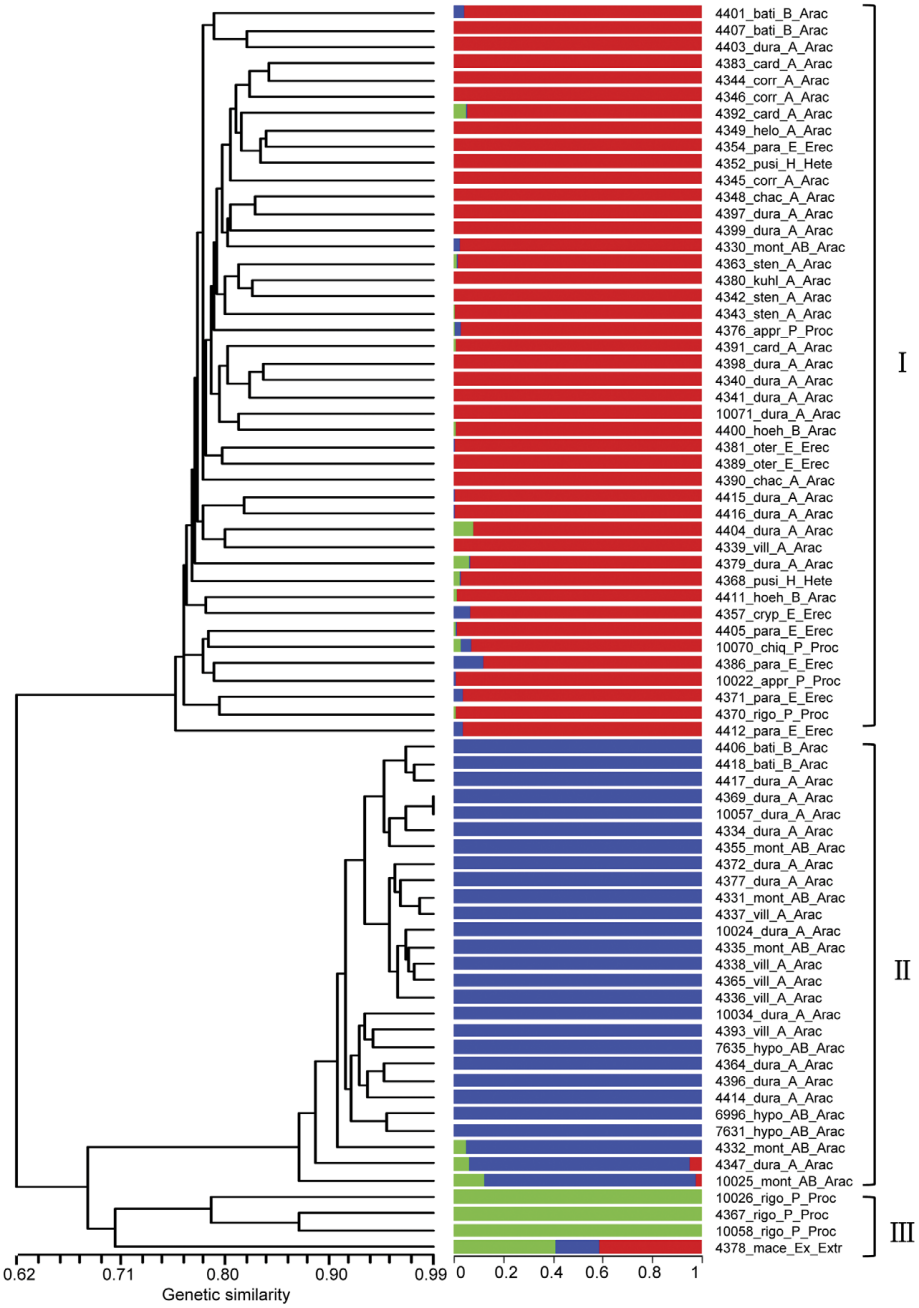
Dendrogram and population structure of 75 wild and cultivated *Arachis* accessions. The 75 *Arachis* accessions were classified into three clusters by structure analysis, I, II, and III, basically corresponding to the phylogenic dendrogram. Red, blue and green corresponds to cluster I, II, and III, respectively. The proportion of each color of the horizontal bar represents the assignment possibilities to the specific cluster. The names of accessions and taxonomical information are given next to the horizontal bars, starting with the accession number followed by an abbreviated form of species name followed by respective genomes and sections. (Abbreviated species names: appr: *A. appressipila*; bati: *A. batizocoi*; card: *A. cardenasii*; chac: *A. chacoense*; chiq: *A. chiquitana*; corr: *A. correntina*; cryp: *A. cryptopotamica*; dura: *A. duranensis*; helo: *A. helodes*; hoeh: *A. hoehnei*; hypo: *A. hypogaea*; kuhl: *A. kuhlmannii*; mace: *A. macedoi*; mont: *A. monticola*; oter: *A. oteroi*; para: *A. paraguariensis*; pusi: *A. pusilla*; rigo: *A. rigonii*; sten: *A. stenosperma*; vill: *A. villosa*; Abbreviated section names: Arac: *Arachis*; Hete: *Heteranthae*; Proc: *Procumbentes*; Erec: *Erectoides*; Extr: *Extranervosae*).

The population structure of the 75 *Arachis* accessions, representing 20 species belonging to 5 sections, was analyzed using 136 SSR markers and the model-based software STRUCTURE. The structure analysis was performed by setting the possible number of groups (*k*), ranging from 1 to 10, with 5 replications for each *k*. The LnP(D) value increased continuously with the increase of *k* and peaked at *k*  = 3 (Figure S1). Accordingly, the 75 *Arachis* accessions were classified into three clusters. Cluster I comprised 45 accessions from the *Arachis*, *Procumbentes*, *Heteranthae* and *Extranervosae* sections. There were 30 accessions from *Arachis* (11 *A. duranensis*, 3 *A. cardenasii*, 3 *A. correntina*, 3 *A. stenosperma* s, 2 *A. batizocoi*, 2 *A. chacoense*, 2 *A. hoehnei*, 1 *A. helodes*, 1 *A. kuhlmannii*, 1 *A. villosa* and 1 *A. monticola*), 8 accessions from *Erectoides* (5 *A. paraguariensis*, 2 *A. oteroi*, and 1 *A. cryptotamica*), 4 accessions from *Procumbentes* (2 *A. appressipila*, 1 *A. chiquitana* and 1 *A. rigonii*), 2 accessions from *Heteranthae* (*A. pusilla*), and 1 accession from *Extranervosae* (*A. macedoi*). Cluster II consisted of 28 accessions from *Arachis*, including 13 *A. duranensis*, 5 *A. monticola*, 5 *A. villosa*, 2 *A. batizocoi* and 3 *A. hypogaea* accessions. Cluster III contained 3 accessions, WH10026, WH10058, and WH4367, belonging to the species *A. rigonii* in the section *Procumbentes* (Figure 1). The phylogenic analysis also grouped the 75 *Arachis* accessions into 3 clusters (I, II and III) corresponding to the structure analysis with only one exception; WH4378 was assigned to cluster I in the structure analysis but classified in cluster III in the phylogenic analysis (Figure 1).

**Figure 2:**
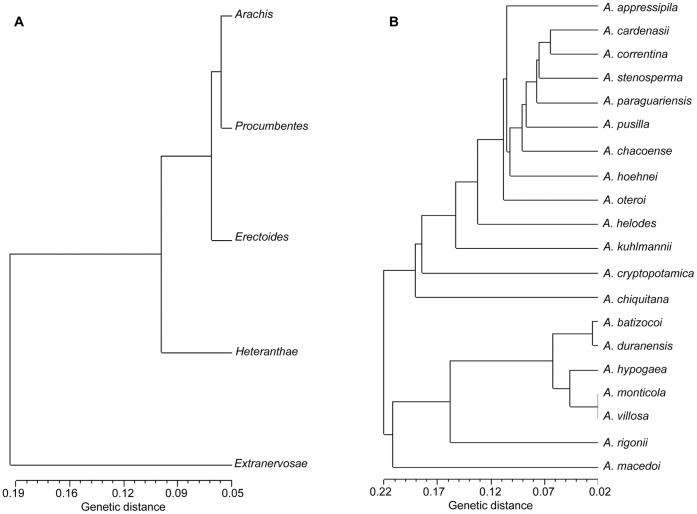
Nei’s distance based dendrogram showing the genetic relations among a) sections and b) species of *Arachis*.

The dendrogram for sections and species of *Arachis* was constructed based on Nei’s distance to analyze pair-wise relationships. *Arachis* and *Procumbentes* were clustered together with a genetic distance of 0.054. *Extranervosae* stood alone, showing a relatively large distance from other sections (*Procumbentes*: 0.162, *Arachis*: 0.164, *Erectoides*: 0.206, *Heteranthae*: 0.245) (Figure 2A). *A. macedoi* (in section *Extranervosae*) was highly differentiated from other species and had the largest distance from *A. cryptopotamica* (0.315; Figure 2B). *A. villosa* and *A. monticola* were the most closely related, with the shortest distance between two species (0.017). Cultivated peanut accessions were clustered with the probable diploid progenitor (*A. duranensis*, AA, distance as 0.079) and tetraploid progenitor (*A. monticola*, AABB, distance as 0.043), as well as with two other wild species from the sections *Arachis*, *A. batizocoi* (BB, distance: 0.099) and *A. villosa* (AA, 0.044) (Figure 2B).

### Oil Content Variation in Wild *Arachis*

**Figure 3:**
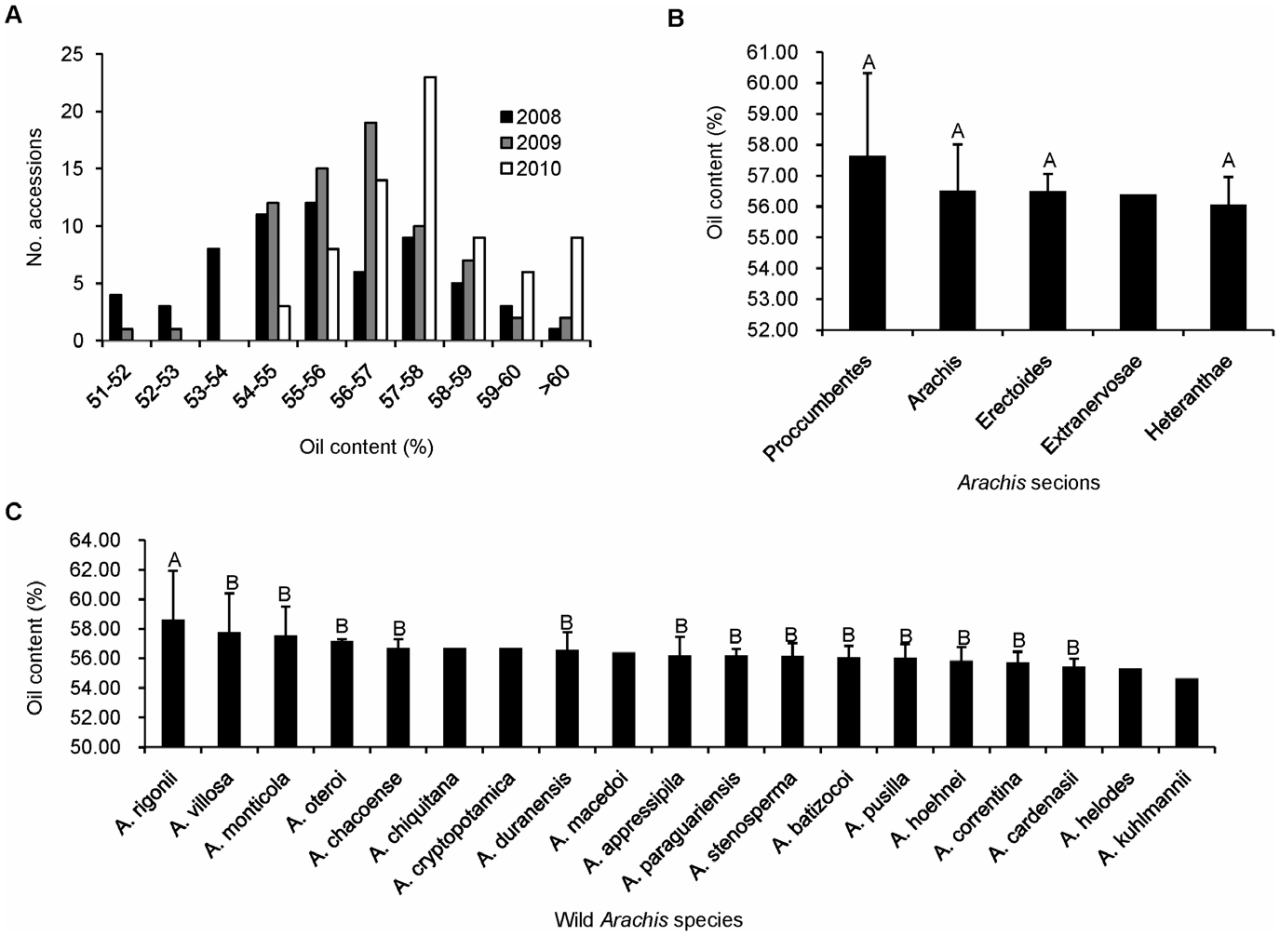
Phenotypic variability of oil content among accessions, species and sections of *Arachis*. a) The distribution of oil content of the 72 wild *Arachis* accessions across three consecutive years from 2008 to 2010; b) Oil content variation among five sections of *Arachis*; c) Oil content variation among twenty species of *Arachis*. The oil content comparisons among sections and species were based on the average value of three years. The capital letters above the bars for the sections and species indicate the significance of difference.

The oil content of the 72 wild *Arachis* accessions was tested over three years, from 2008 to 2010. A significant phenotypic variation of oil content was observed in the 72 wild *Arachis* accessions, ranging from 51.44% to 62.90%, 51.40% to 62.79% and 54.22% to 63.34% in 2008, 2009 and 2010, respectively (Figure 3A). The oil content showed a normal distribution in the 72 wild *Arachis* accessions, and the phenotypic distributions were similar in the three years. The peak oil content distribution in 2010 was slightly higher than those in 2008 and 2009 (Figure 3A) due to the weather. Among the 72 wild *Arachis* accessions, most lines had oil contents ranging from 55% to 58%, and an *A. rigonii* accession (WH10026) had the highest oil contents (62.90%, 62.79% and 61.10%) in all three years. In addition, 7 accessions (WH4347, WH4377, WH10034, WH4330, WH10025, WH4376, and WH4367) not only had oil contents of more than 57% in each season but also had coefficients of variation lower than 0.01.

The average oil content of the wild *Arachis* accessions was compared to assess the influence of species differentiation on trait performance. The accessions from section *Procumbentes* had an average oil content of 57.65% (ranging from 54.31% to 62.26%), higher than that in other sections (Figure 3B). Among the 19 wild *Arachis* species, *A. rigonii* showed the highest average oil content, at 58.62%. *A. kuhlmannii* had the lowest average oil content at 54.56% (Figure 3C). Of the 19 wild species evaluated, 7 had higher oil content than the average of 72 wild *Arachis* accessions (56.69%): *A. chacoense* (56.70%), *A. monticola* (57.57%), *A. villosa* (57.75%), *A. cryptopotamica* (56.69%), *A. oteroi* (57.18%), *A. chiquitana* (56.70%), and *A. rigonii* (58.62%) (Figure 3C).

### Association Analysis of Oil Content in *A. duranensis*

Based on the *Q* model, an association analysis of oil content was performed to determine which SSR alleles of the 23 *A. duranensis* accessions (AA) were most highly correlated with oil content (Table 4). A total of nine alleles were found to be significantly associated with oil content (P<0.01). Four alleles, with sizes of 320 bp, 330 bp, 340 bp and 350 bp, were amplified from the marker PM204. These were significantly associated with oil content, and the 320 bp allele accounted for the largest phenotypic variation in oil content (31.67%; P = 5.43×10^−4^).

**Table 4:** Detailed information of SSR marker alleles showing significant associations with oil content in the accessions of *Arachis duranensis.*

SSR primer	Associated alleles (bp)^a^	P value	Variance (%)^b^	Effects^c^	Variety^d^
PM204	320	0.0005429	31.69	−0.18	WH4416
	330	0.0041	23.94	+1.76	WH4377
	***340***	0.002	26.84	+1.57	WH4377
	***350***	0.0095	20.29	+1.68	WH4377
2A5	270	0.0027	25.58	−0.85	WH4398
	280	0.0078	21.16	+0.03	WH4396
11H1	570	0.0018	28.87	−0.13	WH4416
3B8	***440***	0.0024	26.09	+0.15	WH10034
TC9E8	680	0.0071	21.58	−0.77	WH4416

a Bold and italics are associated SSR alleles exclusively amplified in wild *Arachis* species of section *Arachis*, but not *Arachis hypogaea*.

b Percentage of phenotypic variance explained by the associated SSR allele.

c Oil content changes of the accessions with the presence of associated alleles relative to the absence of associated alleles. Positive value indicates presence of the associated alleles increase oil content, whereas negative value indicates presence of the associated alleles decrease oil content.

d The accessions harboring the associated alleles, which exhibited the highest oil content for the positive effects, and the lowest oil content for the negative effects.

The accessions were grouped based on the presence or absence of associated alleles to assess the combined effect of the associated alleles on oil content (Table 4). Among the nine associated alleles, five were linked to increased oil content. The 330 bp allele amplified from the marker PM204 correlated with the highest increase in phenotypic oil content, likely enhancing the oil content by approximately 1.76%. The remaining four alleles were linked to decreases in oil content. The 270 bp allele amplified from marker 2A5 correlated with the largest decrease in oil content, likely decreasing the oil content by 0.88%. Out of the nine alleles associated with oil content, six alleles were shared between *A. hypogaea* and its wild relatives; four of six alleles were linked to decreased oil content and two alleles were linked with increased oil content. The remaining three alleles were exclusively amplified in wild relatives but not in *A. hypogaea,* and all were linked to increased oil content (Table 4).

## Discussion

### Allele Diversity and Genetic Differentiation among Species and Sections within the Genus *Arachis*

The evaluation of the genetic diversity in *Arachis* germplasm is crucial for the efficient exploitation of the valuable alleles present in wild species during cultivated peanut improvement, which has been demonstrated in several previous studies [23], [24], [25]. In the past, only a few dozen markers were used to assess genetic diversity. In the present study, we investigated the genetic diversity of 72 wild *Arachis* and 3 *A. hypogaea* accessions using 136 genome wide SSR markers. A total of 944 alleles (6.94 alleles per marker and a Shannon-Weaver diversity index of 0.4130) were detected in the wild *Arachis* species. The number of alleles was higher than that in *A. hypogaea* (Table 1), indicative of the low polymorphism in *A. hypogaea* [25], [26]. The large difference of genetic diversity between the cultivated peanut accessions and the wild species can likely be attributed to (a) domestication events that greatly reduced genetic diversity of the cultivated peanut [10], (b) limited natural gene exchange between diploid wild species and cultivated peanut due to genomic rearrangement during the polyploidization event [9], [27], and (c) the founder effect in improvement of cultivated peanut caused by the use of few elite breeding lines and little exotic germplasm in breeding programs [11]. This finding illustrates the potential in introgressing genetic diversity from wild *Arachis* to broaden the genetic base of cultivated peanut breeding. *A. duranensis* of the section *Arachis* exhibited the highest diversity among the 19 wild *Arachis* species studied (Table 1), indicating the great potential of its use in cultivated peanut improvement [8].

The presence of alleles that are unique to a specific population is an effective indicator of genetic differentiation in populations [22], [28]. In the present study, 75 SSR markers amplified a total of 129 unique species-specific alleles in the genus *Arachis* (Table 1). Different numbers of unique alleles were observed in different species, reflecting the different levels of speciation and diversity of these species [22]. A significant level of genetic differentiation among sections and species of wild *Arachis* (P<0.001, Table 2) was demonstrated by AMOVA, which confirmed the distinct nature of different *Arachis* species. *A. rigonii,* of the section *Procumbentes*, originated in Eastern Bolivia and northern Argentina, the origin center of wild *Arachis* [29], and contained the largest number of unique alleles (75, Table 1). This suggests that the alleles specific to *A. rigonii* are likely native variants that were lost after natural selection or domestication events. It was previously observed that the majority of Arachis species harboring species-specific alleles had originated in Brazil [30]. These unique alleles would be useful markers for efforts to broaden the genetic base of breeding pools and enhance economically important traits, i.e., by introgressing the specific alleles into the cultivated peanut.

### Relationships among the 75 *Arachis* Accessions

*Arachis* species are highly differentiated from each other, and the hybrids between most species are sterile [1]. It is easier to transfer valuable alleles between species that are closely related. Thus, the evaluation of the genetic relationships among various *Arachis* species is necessary for the successful and efficient exploitation of the genetic diversity that exists within this genus. The present study assessed the relationships among 75 accessions representing 20 species from the sections *Arachis*, *Erectoides*, *Extranervosae*, *Heteranthae* and *Procumbentes*. The 75 *Arachis* accessions were generally grouped into 3 clusters corresponding to their species, sections and genome types in a structural analysis, and this grouping correlated nearly perfectly with the classification based on phylogenic analysis (Figure 1). Only one accession (WH4378) was assigned to different clusters in the structural analysis (cluster I) and the phylogenic analysis (cluster III). This could be due to the statistical bias of the model-based structure analysis, as WH4378 was the only accession from section *Extranervosae* in this study. Most accessions of *A. monticola* (AABB) were clustered together with *A. hypogaea* (AABB) (Figure 1), indicating the high similarity between these two tetraploid species. This supports the hypothesis that *A. monticola* is the direct ancestor of *A. hypogaea* [3], [4]. The accessions of *A. villosa* (AA) and *A. batizocoi* (BB) were also grouped with the accessions of *A. hypogaea* (Figure 1). This suggests that these two wild diploid species are also closely related to the cultivated peanut. The accessions of *A. diogoi* and *A. batizocoi* showed the least genetic distance from *A. hypogaea* after the presumed diploid progenitors *A. duranensis* and *A. ipaënsis* [30]. This finding indicates that it may be possible to transfer valuable alleles from *A. villosa* and *A. batizocoi* into the cultivated species through direct hybridization. The three *A. rigonii* accessions of section *Procumbentes* exist in a lone cluster (Figure 1), suggesting a high level of differentiation between *A. rigonii* and other species. This is consistent with the finding that *A. rigonii* had the greatest number of species-specific alleles (Table 2). Furthermore, the remaining *Procumbentes* accessions clustered together with the sections *Arachis*, *Erectoides*, and *Heteranthae*, indicating that the genetic variation within this section may be so high that accessions from the same section cannot be clustered together (Table 3) [30]. This pattern also suggests that the species of different sections dispersed in sympatric habitats are most likely clustered together [29].

### Evolutionary Inferences for the Cultivated Peanut

The two *Arachis* species *A. duranensis* (AA) and *A. ipaënsis* (BB) are believed to be the wild diploid progenitors of *A. hypogaea* (AABB), which experienced an interspecific hybridization event followed by chromosome duplication [3], [5]. The wild tetraploid *Arachis* species *A. monticola* (AABB) was proposed to be the direct ancestor of *A. hypogaea* because a high level of similarity between the two species was shown in molecular marker and cytological analyses [3], [4]. Investigation of the genetic differences between the cultivated peanut and its diploid and tetraploid wild relatives is of great importance for inferring the evolutionary history of cultivated peanut. In the present study, we found six evolutionary modes for the alleles of the cultivated peanut. The majority of alleles were represented by evolutionary modes iii, iv and vi (Table 3), indicating that most alleles emerged during the speciation of the progenitor diploid wild *Arachis* species but were lost after the polyploidization and domestication events. More alleles were associated with mode iv than with mode i (Table 3), suggesting that directional selection and the hitchhiking effect played more important roles than did novel mutations in the domestication of the cultivated peanut [31]. This finding implies that many alleles that exist in wild *Arachis* relatives have been lost in the cultivated peanut because of domestication by ancient breeders. The effective utilization of these ancient alleles in wild relatives will be important the improvement of the cultivated peanut.

### The Potential of Wild *Arachis* Germplasm in Enhancing Oil Content in Cultivated Peanut

Wild *Arachis* germplasm has been shown to harbor high-level resistance to foliar and viral diseases, which is not observed in the cultivated peanut [12], [15], [16]. Some wild *Arachis* materials have been used successfully to develop new peanut varieties that are resistant to pests and disease [19], [20], [32]. In the present study, the average oil content of 19 wild *Arachis* species was 56.69%; seven species had oil content higher than 56.69% (Figure 3C). This was significantly higher than the oil content of the cultivated peanut germplasm, which averaged 50.76% over 6390 accessions [33]. These results suggest that the wild *Arachis* accessions are great potential resources for enhancing the oil content of the cultivated peanut, in addition to conferring resistance to pest and disease.

Association analysis is an efficient approach for identifying alleles that are correlated with target traits [34]. In the present study, nine alleles were found to be significantly associated with oil content (P<0.01, Table 4). The SSR marker 2A5 amplified the 270 bp and 280 bp alleles in the wild *Arachis* accessions, which account for 25.58% and 21.16% of the phenotypic variance in oil content, respectively (Table 4). The 240 bp and 250 bp alleles of 2A5 were amplified in the high and low oil content materials of cultivated peanut with a match rate of 88.9% and 95.0%, respectively [35]. These alleles had previously been demonstrated to be associated with oil content, which confirmed the reliability of the association analysis results. These same markers could amplify multiple associated alleles with different effects (Table 4) [35], which may be due to the functional differentiation of orthologous genomic regions among evolutionarily related species [36]. Among the nine associated alleles, the 340 bp and 350 bp alleles from PM204 and the 440 bp allele from 3B8 were amplified exclusively in the accessions of *A. duranensis* and *A. monticola* and not in the accessions of *A. hypogaea* (Table 4). This suggests that these alleles had undergone directional selection or selective hitchhiking that greatly reduced their genetic variability [37]. Three wild species had specific alleles associated with increased oil content (Table 4), and introgression of these alleles into the cultivated peanut may be a useful strategy for improving oil content. The accessions of *A. duranensis* are highly cross-compatible with *A. hypogaea* [1]; two *A. duranensis* accessions, WH4377 and WH10034, are of particular value. WH4377 harbored the 340 bp and 350 bp alleles from PM204, and WH10034 harbored the 440 bp allele from 3B8. Thus, these three alleles correlated with increased oil content could be introgressed into cultivated varieties.

## Materials and Methods

### Plant Materials and Oil Content Phenotyping

A collection of 72 wild *Arachis* accessions was assembled. These accessions represented 19 species from the sections *Arachis*, *Erectoides*, *Extranervosae*, *Heteranthae* and *Procumbentes*. Three widely-grown peanut cultivars (*A. hypogaea*) of China, Zhonghua8, Zhonghua12 and Baisha1016, were also included for comparison with their wild *Arachis* relatives. Detailed information for the 75 *Arachis* accessions is listed in Table S1.

The 72 wild *Arachis* accessions were planted in the Wild *Arachis* Nursery of the Oil Crops Research Institute of the Chinese Academy of Agricultural Sciences in Wuhan, China. Each accession was planted in a single row, with 10 plants in each row, 10 cm between plants within each row and 30 cm between rows. Oil content was tested with the Soxhlet extraction method using fresh dried mature seeds with intact testa harvested between 2008 to 2010.

### SSR Genotyping

Genomic DNA was extracted from young leaves collected from eight to ten plants of each accession using a modified cetyltrimethyl ammonium bromide (CTAB) method [38]. The integrity and quality of the DNA was evaluated on a 1% agarose gel by comparison with known concentrations of uncut lambda DNA standard.

A total of 136 SSR markers from different resources were used to genotype the 75 *Arachis* accessions. SSR markers with the prefixes pPGPseq, pPGSseq, PM, Ah, IPAHM, PMc, Lec, EM, AC, gi, RN, TC, and AHBGS were obtained from the literature [4], [39], [40], [41], [42], [43], [44], [45], [46], [47]. SSR markers with the prefixes XY and POCR were developed by our laboratory [48]. PCR reactions followed the protocol described by Chen et al. [38]. PCR products were visualized on a 6% polyacrylamide gel followed by silver staining. The fragment sizes of the PCR products were estimated by comparison with a 50 bp DNA ladder. Each polymorphic fragment was scored as ‘1′ or ‘0′ according to the presence or absence of amplification.

### Statistical Analysis

#### Genetic and oil content variation

The total number of alleles, number of alleles per marker, and the Shannon-Weaver diversity index were analyzed using the PopGene 1.32 program [49] to evaluate the genetic variability within the wild *Arachis* sections and species and cultivated peanut. Unique alleles were used to evaluate the genetic variants exclusive to the specific section or species. The phenotypic variation of the oil content in the 72 wild *Arachis* accessions and the difference in oil content among sections and species were analyzed using SAS 8.02 [50].

#### AMOVA

To investigate the genetic differentiation among the 72 wild *Arachis* accessions, analysis of molecular variance (AMOVA) was performed using the software package Arlequin 3.1 [51] with 1,000 permutations and the sum of squared size differences as the molecular distance.

#### Population structure and phylogenic analyses

The population structure of the 75 *Arachis* accessions was analyzed using 136 SSR markers and the program STRUCTURE 2.2 [52]. Five independent simulations were performed for values of *k* (the number of groups) ranging from 1 to 10. For each simulation, 10,000 iterations before a burn-in length of 10,000 MCMC (Markov Chain Monte Carlo) replications were conducted with the admixture and related frequency models. The optimal *k* value was determined by the posterior probability [LnP(D)]. Accessions were assigned to a corresponding group based on their maximum membership probabilities, as described by Remington et al. [53]. A UPGMA dendrogram was constructed to better depict the relationship among the 75 *Arachis* accessions using NTSYS 2.0 [54]. Based on Nei’s (1973) genetic distance [55], a dendrogram of the sections and species of *Arachis* was also constructed using PowerMarker 3.51 [56].

#### Association analysis of oil content

A complex genetic structure may lead to a high possibility of false positives in association mapping [57]. Because wild *Arachis* species are highly differentiated [1], [2], 23 accessions of *A. duranensis* were selected to perform an association analysis to investigate SSR alleles highly correlated with oil content. The mean values of oil content over three consecutive years rather than values in a single year were used to increase the statistical power of the association mapping. The *Q* model, controlling the population structure of 23 *A. duranensis* accessions derived from the structure analysis, was used to perform the GLM procedure in the package TASSEL 2.1 [58]. The SSR allele is significantly associated with oil content when the P value is less than 0.01. The R^2^ value indicates the percentage of phenotypic variance explained by the associated allele. Allele effects on oil content were estimated based on the difference in the oil content of accessions with the allele compared to accessions without the allele. A positive value indicates that the associated allele increases oil content, whereas a negative value indicates the associated allele decreases oil content.

## Supporting Information

Figure S1 **Estimation of LnP(D) in the 75 accessions of** ***Arachis*** **.** The bar indicates standard deviation.
(PPT)

Table S1 **List of 75** ***Arachis*** **accessions representing 20 species and 5 sections.**
(XLS)
