# Supplementary figures and images for: Abundant Microsatellite Diversity and Oil Content in Wild Arachis Species
Source: PLoS One. 2012 Nov 20;7(11):e50002. doi: 10.1371/journal.pone.0050002 (PMC3502184; doi:10.1371/journal.pone.0050002)

## Slide 1
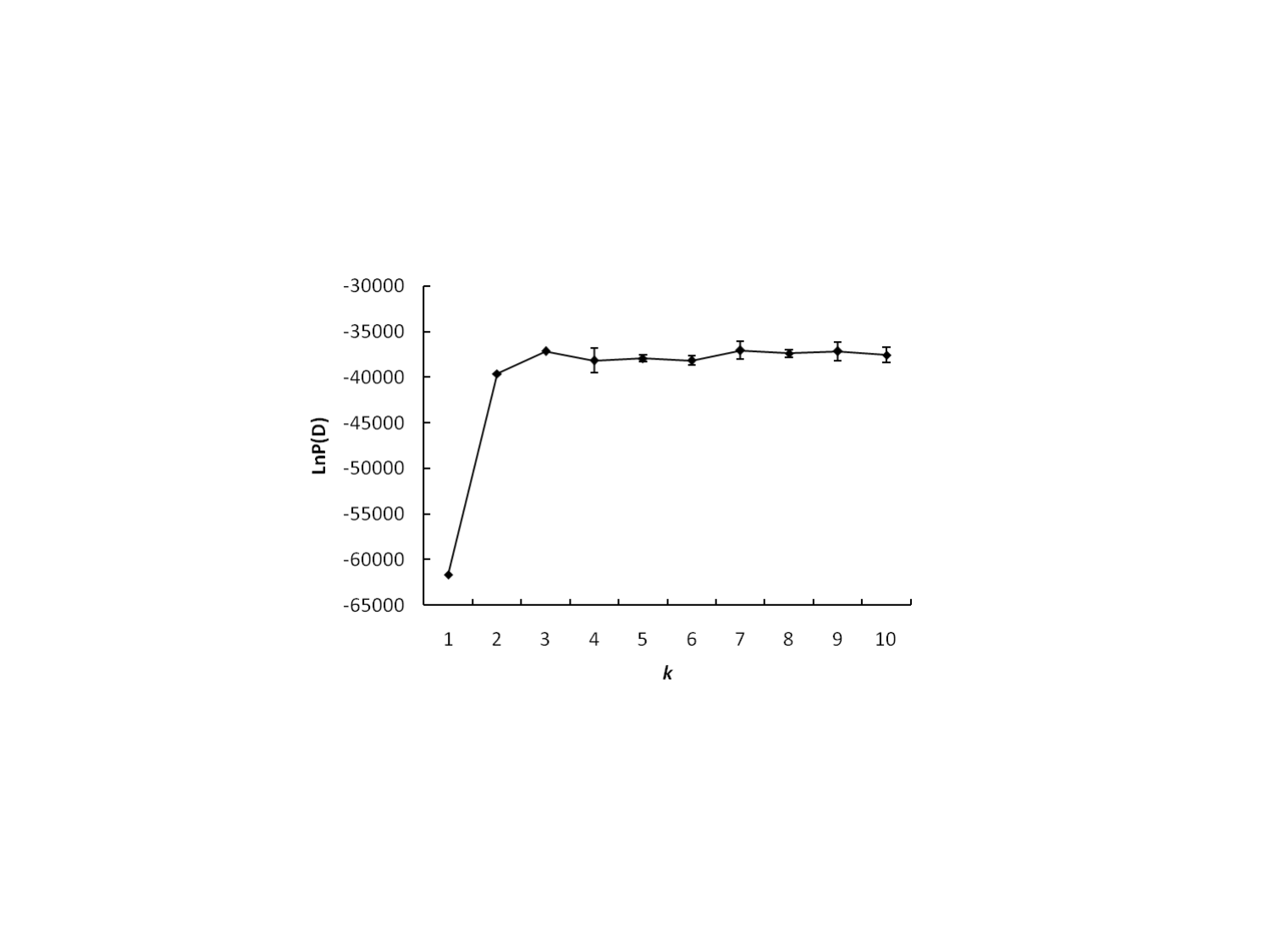

Supplement: Figure S1 — Estimation of LnP(D) in the 75 accessions of Arachis . The bar indicates standard deviation. (PPT) [file pone.0050002.s001.ppt]
